# The Nuclear Guanine Nucleotide Exchange Factors Ect2 and Net1 Regulate RhoB-Mediated Cell Death after DNA Damage

**DOI:** 10.1371/journal.pone.0017108

**Published:** 2011-02-23

**Authors:** Melissa C. Srougi, Keith Burridge

**Affiliations:** 1 Department of Cell and Developmental Biology, University of North Carolina, Chapel Hill, North Carolina, United States of America; 2 Lineberger Comprehensive Cancer Center, University of North Carolina, Chapel Hill, North Carolina, United States of America; University of Birmingham, United Kingdom

## Abstract

Commonly used antitumor treatments, including radiation and chemotherapy, function by damaging the DNA of rapidly proliferating cells. However, resistance to these agents is a predominant clinical problem. A member of the Rho family of small GTPases, RhoB has been shown to be integral in mediating cell death after ionizing radiation (IR) or other DNA damaging agents in Ras-transformed cell lines. In addition, RhoB protein expression increases after genotoxic stress, and loss of RhoB expression causes radio- and chemotherapeutic resistance. However, the signaling pathways that govern RhoB-induced cell death after DNA damage remain enigmatic. Here, we show that RhoB activity increases in human breast and cervical cancer cell lines after treatment with DNA damaging agents. Furthermore, RhoB activity is necessary for DNA damage-induced cell death, as the stable loss of RhoB protein expression using shRNA partially protects cells and prevents the phosphorylation of c-Jun N-terminal kinases (JNKs) and the induction of the pro-apoptotic protein Bim after IR. The increase in RhoB activity after genotoxic stress is associated with increased activity of the nuclear guanine nucleotide exchange factors (GEFs), Ect2 and Net1, but not the cytoplasmic GEFs p115 RhoGEF or Vav2. Importantly, loss of Ect2 and Net1 via siRNA-mediated protein knock-down inhibited IR-induced increases in RhoB activity, reduced apoptotic signaling events, and protected cells from IR-induced cell death. Collectively, these data suggest a mechanism involving the nuclear GEFs Ect2 and Net1 for activating RhoB after genotoxic stress, thereby facilitating cell death after treatment with DNA damaging agents.

## Introduction

Current cancer treatment modalities include the use of radio- and chemotherapeutic agents that damage DNA. In healthy cells, exposure to these agents activates mechanisms to either repair sites of DNA damage or if the damage is irreparable, to activate the cell death machinery. However in cancer cells, prolonged exposure to these agents can lead to resistance, which is a common clinical problem. Therefore, understanding the pathways whereby cancer cells respond to DNA damage may provide insights into how tumors will respond to therapy and circumvent possible resistance mechanisms. Members of the Rho family of GTPases have been well established as playing key roles in the dynamic regulation of the actin cytoskeleton. It is also clear that they coordinate a wide variety of diverse cellular processes important in tumorigenesis including gene expression, cell proliferation and survival [1]. Although a number of studies indicate growth stimulatory roles for RhoA and RhoC in cancer, the exact role of RhoB in tumorigenesis is still being defined. Despite sharing 86% homology to RhoA, in some cell types RhoB exerts more of a tumor-suppressor role, as loss of RhoB is associated with various types of human tumors [2], [3], [4] and an increase in metastatic potential [5].

RhoB has a short protein half-life (∼2 h) and its protein expression is readily inducible upon exposure to a wide-variety of biological stimuli including growth factors such as platelet-derived growth factor (PDGF), epidermal growth factor (EGF) as well as transforming growth factor-β (TGF-β) [6], [7], [8]. In addition to growth factors, RhoB is known to be up-regulated in response to DNA damaging agents (UV, cisplatin [9]), and cellular stress [10]. In this context, Ras-induced loss of RhoB protein reduces the sensitivity of transformed cells to genotoxic agents *in vitro* [11]. Furthermore, targeted deletion of RhoB in mouse embryonic fibroblasts (MEFs) confers cellular resistance of transformed cells to γ-irradiation (IR) or taxol [12].

With predominant roles for Rho proteins in a number of cellular processes, it is not surprising that the activity of these molecules is tightly regulated. Rho protein function is modulated by three main classes of regulatory molecules, which control the transition of Rho from an inactive GDP-bound form to an active GTP-bound form. GTPase-activating proteins (GAPs) decrease Rho protein activity by stimulating their intrinsic GTP hydrolysis activity [13]. Guanine nucleotide-dissociation inhibitors (GDIs), sequester GDP-bound Rho in the cytosol keeping them inactive. Conversely, guanine nucleotide exchange factors (GEFs) increase the activity of Rho proteins by promoting the exchange of GDP for GTP. As important as Rho-protein signaling is in contributing to the cancer cell phenotype, no GTPase-defective mutants have been found in human tumors to date. However, as spatio-temporal activators of Rho, GEFs represent a large class of proteins where their altered regulation and/or localization can have a dramatic impact on tumor progression and possibly tumor response to therapy.

Here, we report that RhoB is activated in human breast and cervical cancer cell lines shortly after treatment with agents that cause DNA damage. Loss of RhoB using shRNA caused partial resistance to IR-induced cell death and inhibited the initiation of apoptotic signaling events. Further investigation revealed that the increase in active RhoB was caused by the specific activation of nuclear GEFs, epithelial cell transforming sequence 2 (Ect2) and neuroepithelial transforming gene 1 (Net1). Inhibition of Ect2 and Net1 decreased RhoB activation, attenuated JNK phosphorylation and induction of the pro-apoptotic protein Bcl-2 interacting mediator of cell death (Bim) causing cellular resistance to IR. These studies identify and highlight the regulatory molecules Ect2 and Net1 that govern RhoB activity after DNA damage in human cancers, and they may be important predictors in tumor response to radio- and chemotherapeutic agents.

## Results

### DNA damage activates RhoB

**Figure 1:**
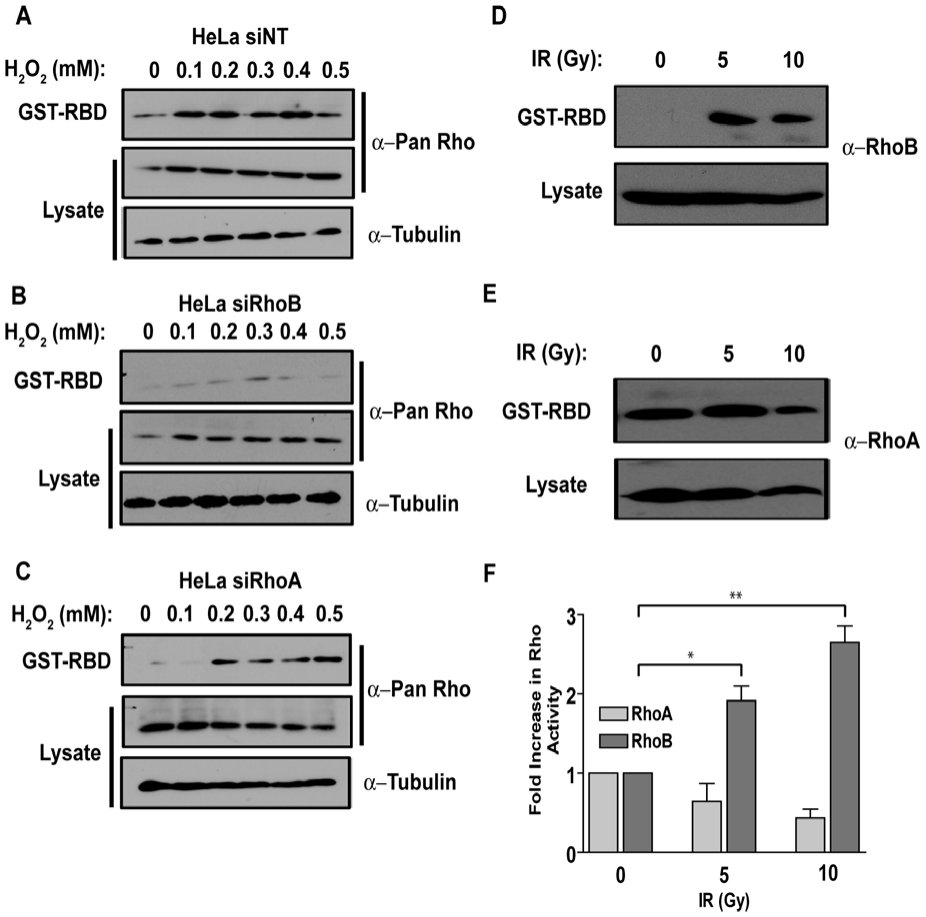
Genotoxic stress increases GTP-bound RhoB. HeLa cells were transfected with (A) an siRNA non-targeting sequence (siNT); (B) an siRNA sequence targeting RhoB (siRhoB); or (C) an siRNA sequence targeting RhoA (siRhoA). 72 h after transfection, cells were treated with increasing doses of H_2_O_2_ for 20 min, then processed for active GTP-bound Rho using a GST-RBD pulldown and blotted for pan-Rho. Lysates served as loading controls and were blotted for Rho or α-Tubulin as indicated. MCF-7 cells were mock-treated or irradiated with 5 or 10 Gy. Active GTP-Rho was determined with GST-RBD pulldowns within 15 min after IR. (D) shows RhoB; (E) shows RhoA. (F) Quantification by densitometry of immunoblots in *(D)* and *(E)* (*, *p*≤0.005; **, *p*≤0.001).

RhoB protein levels are readily inducible upon exposure to genotoxic agents, many of which generate reactive oxygen species (ROS). We therefore wanted to determine if ROS-induced DNA damage specifically alters the levels of GTP-bound, active RhoB. To accomplish this, HeLa cells were transfected with a non-targeting siRNA (siNT), an siRNA targeted to RhoB (siRhoB), or an siRNA targeted to RhoA (siRhoA). The specificity and efficacy of each siRNA was evaluated by immunoblot (Fig. S1A). HeLa siNT, HeLa siRhoB and HeLa siRhoA cells were then treated with H_2_O_2_ for 20 min, which is sufficient to create DNA strand breaks [14] and examined for total Rho activation thereafter using an antibody containing a common epitope for all three Rho proteins (RhoA, RhoB, and RhoC). H_2_O_2_-treated HeLa siNT and parental HeLa cells exhibited an overall increase in total Rho activity (Fig. 1A and Fig. S1B). However, in HeLa cells transfected with an siRNA targeted to RhoB, very little Rho activation was observed after H_2_O_2_ exposure (Fig. 1B). This was also noted in H_2_O_2_-treated parental HeLa cell lysates using an antibody specific for RhoB (Fig. S1C) In contrast to RhoB, suppression of RhoA protein levels by siRNA did not prevent Rho activation after H_2_O_2_ treatment (Fig. 1C). These data suggest that the majority of active, GTP-bound Rho was attributed primarily to RhoB activation (Fig. 1B) as there was only a modest increase in active RhoA at higher H_2_O_2_ exposure (Fig. S1D).

To determine if other types of DNA damaging agents could activate RhoB, or if the effects from the direct administration of H_2_O_2_ were agent specific, we extended our studies to incorporate the use of ionizing radiation, as it is commonly used for the treatment of solid tumors, including those of the breast. Many studies implicating RhoB in cell death after γ-IR and other damaging agents were performed in Ras-transformed cells. We therefore wanted to further explore the role of RhoB activation in a non-Ras transformed cellular system and utilized the breast cancer epithelial cell line, MCF-7 [15], which contains endogenous RhoB. MCF-7 cells were irradiated with 5 and 10 Gy respectively and RBD pulldowns performed to detect active GTP-bound Rho proteins. There was an increase in RhoB activity immediately (within 15 min) following IR that was maintained up to 5 h afterwards (Fig. 1D, 1F and data not shown). Furthermore, an increase in RhoB activity was also observed after treatment with the pyrimidine analog, 5-fluorouracil (5-FU) (Fig. S2A and Materials and Methods S1). The increase in RhoB-GTP levels was specific as the levels of RhoA-GTP under identical conditions remained unaltered (Fig. 1E and 1F). These studies suggest that GTP-bound RhoB levels, but not RhoA levels, increase after exposure to IR and other DNA damaging agents.

### RhoB activity is required for IR-induced cell death in breast cancer cells

**Figure 2:**
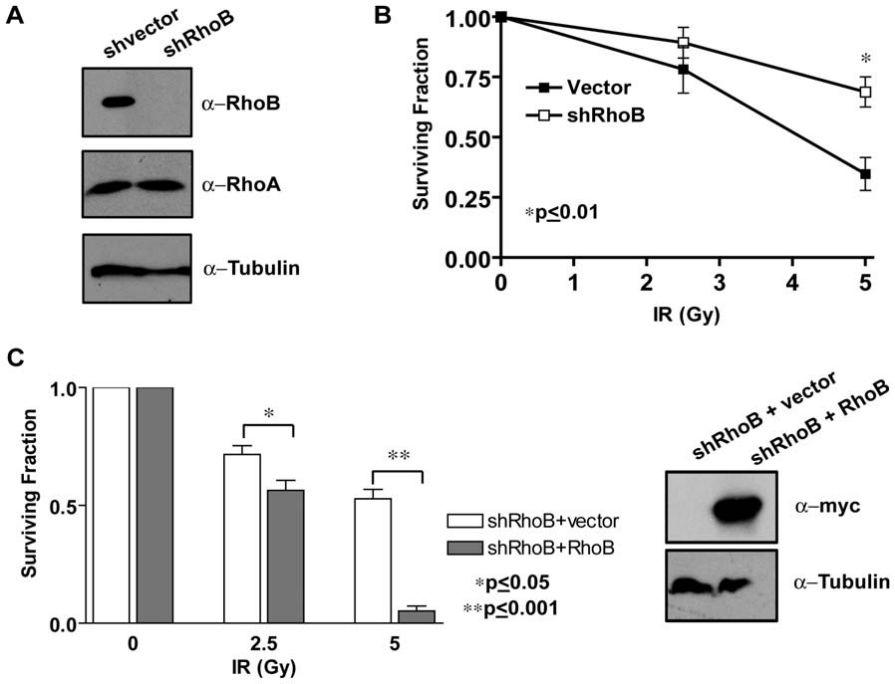
Knock-down (KD) of RhoB confers cellular resistance to IR in human breast cancer cells. (A) Immunoblots showing RhoB, RhoA and α-Tubulin protein levels from whole cell extracts of MCF-7-shvector and MCF-7-shRhoB cell lines. (B) MCF-7-shvector and MCF-7-shRhoB cells were mock-treated or irradiated with 2.5 Gy or 5 Gy and colony forming ability (CFA) was determined approximately 14–20 days after IR. (C) Ectopic re-expression of RhoB sensitizes cells to IR. (*left panel*) MCF-7-shRhoB cells expressing either vector alone or shRNA-resistant wild-type (wt) myc-RhoB were mock-treated or irradiated with 2.5 Gy or 5 Gy and CFA determined 14–20 days after treatment (*, *p*<0.001; **, *p*≤0.05). (*right panel*) Immunoblot showing the re-expression of an shRNA resistant wt myc-RhoB in MCF-7-shRhoB cells.

RhoB activation after IR occurs quickly and robustly (Fig. 1D and 1F), but does this activation have any functional consequences for cellular survival? To test this hypothesis, endogenous RhoB expression was suppressed using a short-hairpin RNA to RhoB. MCF-7 cells stably transfected with RhoB-shRNA (MCF-7-shRhoB) showed a marked reduction in RhoB protein levels compared with MCF-7 cells infected with the shRNA vector alone (MCF-7-shvector) (Fig. 2A). Importantly, knock-down (KD) of RhoB did not affect RhoA protein levels (Fig. 2A). We first examined the effects of loss of RhoB protein on cellular survival after IR. Clonogenic assays were performed on MCF-7-shvector and MCF-7-shRhoB cells that were either mock-treated or irradiated at 2.5 and 5 Gy. No significant differences in survival were noted at sub-lethal doses of IR, however, the survival difference between these two cell lines was readily apparent at higher radiation exposures (Fig. 2B). MCF-7-shRhoB cells were statistically more resistant to 5 Gy than their genetically matched MCF-7-shvector counterparts (Fig. 2B). Similar findings were observed after 5-FU treatment (Fig. S2B). Furthermore, radio-resistance could be reverted in MCF-7-shRhoB cells upon ectopic re-expression of an shRNA-resistant wild-type myc-RhoB construct (Fig. 2C). Collectively, these data suggest that RhoB plays a role in IR-induced cell death.

### The nuclear GEFs Ect2 and Net1 are activated following IR

Our data suggest that RhoB is activated after the DNA damaging agents H_2_O_2_ and IR, and that RhoB is partially required for cell death after IR. However, much of the upstream regulatory processes that govern the control of RhoB activity following DNA damage have not been elucidated. Our laboratory and others have demonstrated that low doses of ROS can directly activate RhoA in fibroblasts and human endothelial cells [16], [17]. This direct activation requires two critical cysteine residues located at amino acid positions 16 and 20 within the phosphoryl binding loop of the protein [17]. Since the redox sensitive motif in RhoB is identical to RhoA, we wanted to determine if RhoB was modulated in a similar manner. Surprisingly, we found that the cysteine residues within the phosphoryl binding loop of RhoB are not sufficient to modulate protein activity after ROS treatment (Fig. S3A). This suggested to us a unique pathway of regulation which differs from RhoA, and is likely mediated by the classical regulatory proteins - GEFs and GAPs.

**Figure 3:**
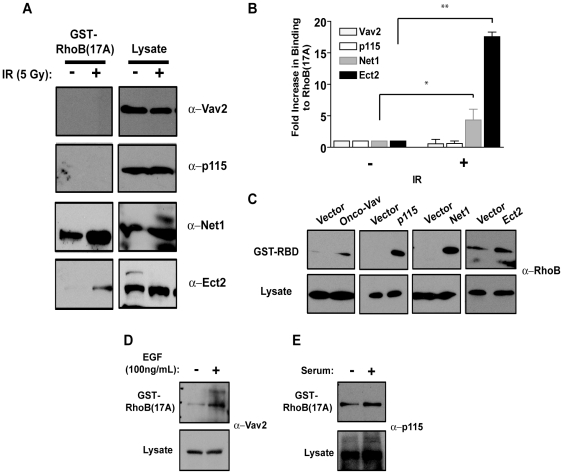
The nuclear GEFs Ect2 and Net1 are activated after IR. (A) MCF-7 cells were mock-treated or irradiated with 5 Gy and pulldowns performed with GST-RhoB(17A) to identify active GEFs. (B) Quantification of blots in *(A)* as described in *Materials and Methods* (*, *p*≤0.05; **, *p*<0.001). (C) MCF-7 cells were transfected with constructs to the indicated GEFs. 24 h after transfection, GST-RBD pulldowns were performed to detect active RhoB. (D and E) MCF-7 cells were serum starved overnight and treated with (D) 100 ng/mL of EGF for 1 h or (E) treated with serum-containing medium for 1 h and pulldowns performed with GST-RhoB(17A). Immunoblots were probed with an antibody to (D) Vav2 or (E) p115 RhoGEF.

To determine which GEFs were responsible for the upstream activation of RhoB after DNA damage, we performed precipitation assays with the nucleotide free RhoB mutant, RhoB(17A). Our laboratory has previously validated the use of this assay for the specific isolation of activated GEFs, which bind with high affinity to the nucleotide free state [18], [19]. MCF-7 cells were irradiated with 5 Gy and pulldowns were performed with GST-RhoB(17A) immediately after IR. Immunoblots of the precipitates were then probed with antibodies to various GEFs to determine which were activated based on their association with the nucleotide-free mutant. We narrowed down our search based on GEF subcellular localization. Since RhoB is activated shortly (∼15 min) after DNA damage, we speculated that the signals responsible for its activation were connected directly to DNA damage signaling pathways. We therefore focused our attention on the nuclear GEFs, Ect2 and Net1. For comparison, we also examined two well-characterized cytoplasmic GEFs; Vav2, which has been shown to exchange upon RhoB [20] and p115 RhoGEF. From these studies we found no activation of the cytoplasmic GEFs, Vav2 and p115 RhoGEF either before or after IR as indicated by binding to the nucleotide-free mutant of RhoB (Fig. 3A). In addition, treatment with H_2_O_2_ did not significantly increase Vav2 or p115 activity (Fig. S3B and S3C) although it caused an increase in RhoB-GTP levels (Fig. S1C). In contrast, both Net1 and Ect2 were activated ≥5 fold after IR (Fig. 3A and 3B). These increases in Ect2 and Net1 activity were also noted after exposure to 5-FU and H_2_O_2_ (Fig. S3B-E).

To ensure that the lack of activation of Vav2 and p115 RhoGEF after IR were not simply due to an inability of these GEFs to exchange upon RhoB, we overexpressed these GEFs in MCF-7 cells and measured RhoB activation. Expression constructs for Vav2, p115 RhoGEF, Net1 and Ect2 were each individually overexpressed in MCF-7 cells and GST-RBD pulldowns performed 24 h following transfection. Overexpression of all tested GEFs resulted in increased RhoB-GTP levels, suggesting that the lack of GEF activation after IR was not due to an inability of the GEFs to facilitate guanine nucleotide exchange upon RhoB (Fig. 3C). Furthermore, stimulation of cells with EGF increased Vav2 activity as demonstrated previously [21] (Fig. 3D) and the addition of serum increased p115 RhoGEF activity (Fig. 3E). Collectively, these data indicate that the nuclear GEFs Ect2 and Net1, but not the cytoplasmic GEFs p115 RhoGEF and Vav2 are activated after IR.

### Ect2 and Net1 are responsible for activating RhoB and leading to cell death after IR

**Figure 4:**
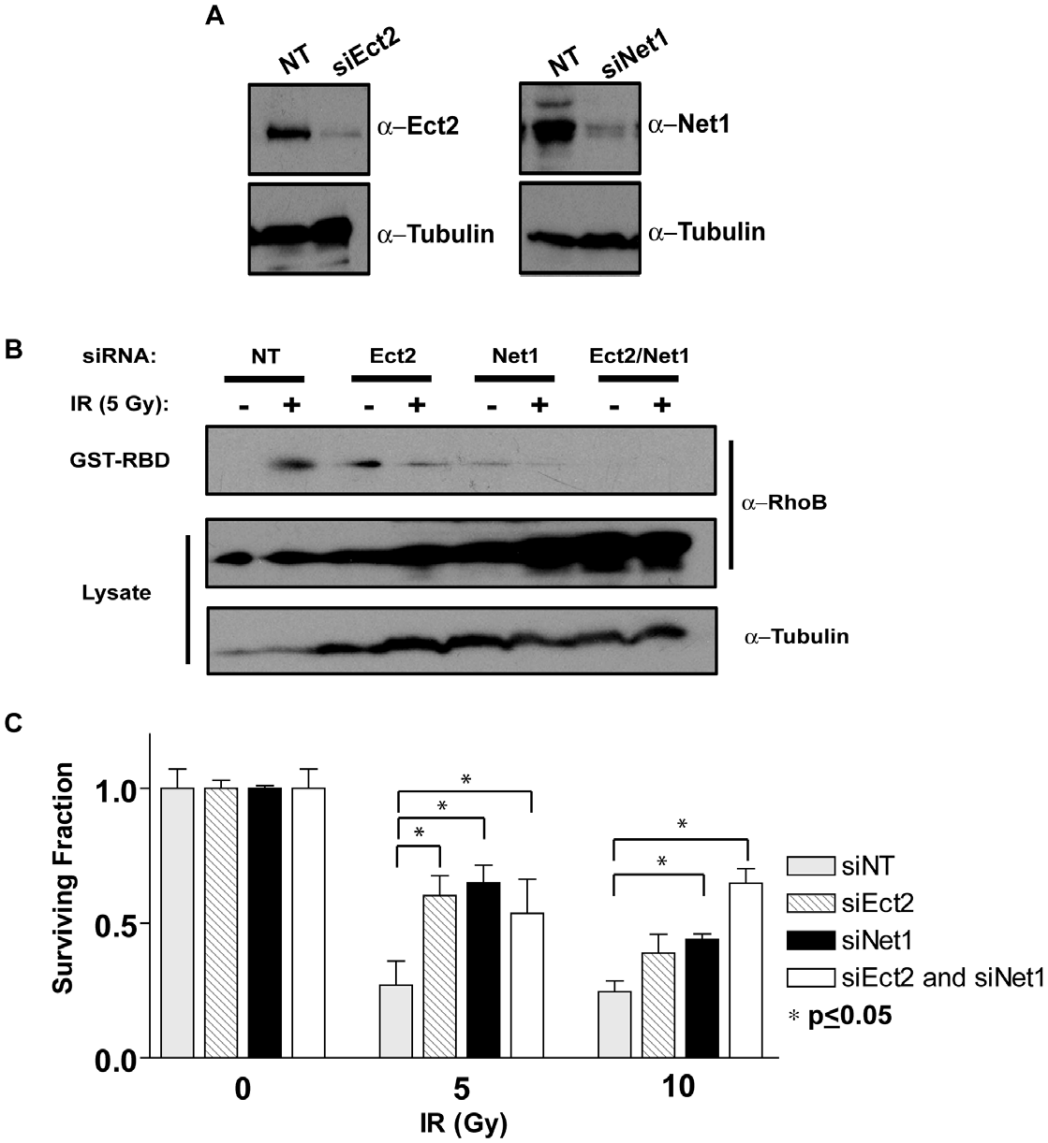
Knock-down of Ect2 and Net1 suppresses RhoB activation and cell death after IR. (A) Immunoblots of Ect2 and Net1 protein levels 72 h after transfection with siRNA oligos targeted to KD Ect2 and Net1, respectively. (B) KD of Ect2 and Net1 suppresses RhoB activation after IR. MCF-7 cells were transfected with a non-targeting oligo (siNT), or oligos targeted to Ect2 (siEct2) or Net1 (siNet1) singly, or a combination of Ect2 and Net1 (siEct2/Net1). 72 h after transfection, cells were mock-treated or irradiated with 5 Gy and GST-RBD pulldowns performed to detect active, GTP-bound RhoB. (C) KD of nuclear GEFs Ect2 and Net1 protects breast cancer cells from radiation-induced lethality. MCF-7 cells were transfected with a non-targeting siRNA or siRNAs to Ect2 and Net1 singly or in combination. 72 h after transfection, cells were mock-treated or irradiated with 5 Gy or 10 Gy then assayed for CFA approximately 14–20 days later.

The specific roles of Ect2 and Net1 were then examined as to their effect on RhoB activation after exposure to IR. MCF-7 cells were transfected with an siRNA targeted to either Ect2 (siEct2), Net1 (siNet1), both (siEct2/Net1), or a non-targeting (siNT) control. KD of steady state protein levels was achieved using either siRNA to Ect2 or Net1 (Fig. 4A). Using this system, MCF-7 cells were irradiated and assayed for RhoB activity. As previously demonstrated, RhoB activity increased after IR in MCF-7 siNT cells (Fig. 4B). However, in siEct2/Net1 cells, RhoB activity was not increased after IR in comparison to the non-targeting control (Fig. 4B). Individual KD of Ect2 and Net1 alone was able to diminish some RhoB activity after IR, but was not as effective as knocking down both GEFs simultaneously (Fig. 4B). These data suggest that IR-induced increases in RhoB activity are a result of both Ect2 and Net1 activation.

It has previously been shown that RhoB is necessary for mediating cell death in various cancer cells [22], and we have shown that it is similarly required for IR-induced cell death in MCF-7 cells (Fig. 2B). Since KD of Ect2 and Net1 were sufficient to suppress RhoB activity after IR, we wanted to test whether these cells would also exhibit cellular resistance to IR in a manner analogous to the loss of RhoB (Fig. 2B). MCF-7 cells were transfected with the indicated siRNAs and 72 h after transfection the cells were irradiated and colony forming ability (CFA) assessed 14 days after treatment. Because Ect2 has been shown to be required for cell proliferation [23], it is important to note that under the conditions used in these studies, transient Ect2 KD had only modest effects on cell proliferation. Mock-irradiated cells exhibited normal CFA, which decreased approximately 80% after irradiation. Single knock-down of Ect2 or Net1 significantly increased CFA at 5 Gy, ∼50% compared with control levels (Fig. 4C). In irradiated cells that were transfected with siRNA targeting both GEFs (e.g. siEct2/Net1) there was a significant increase in CFA at 5 Gy and 10 Gy compared with the siRNA control, 50% and 60% respectively. Furthermore, knocking-down both GEFs increased survival at 10 Gy compared to each single KD alone (Fig. 4C). However, this increase in survival in Ect2 and Net1 KD cells was not as protective as KD of the GTPase itself (Fig. 2B) possibly due to the efficiency of KD (stables *vs.* transients) or other GEFs that may also be involved in the response to DNA damage. Collectively, these data suggest that Ect2 and Net1 play a role in RhoB-mediated cell death as loss of both GEFs decreased RhoB activity and partially protected cells from IR-induced cell death.

### Loss of Ect2 and Net1 suppresses IR-induced apoptotic signaling pathways

**Figure 5:**
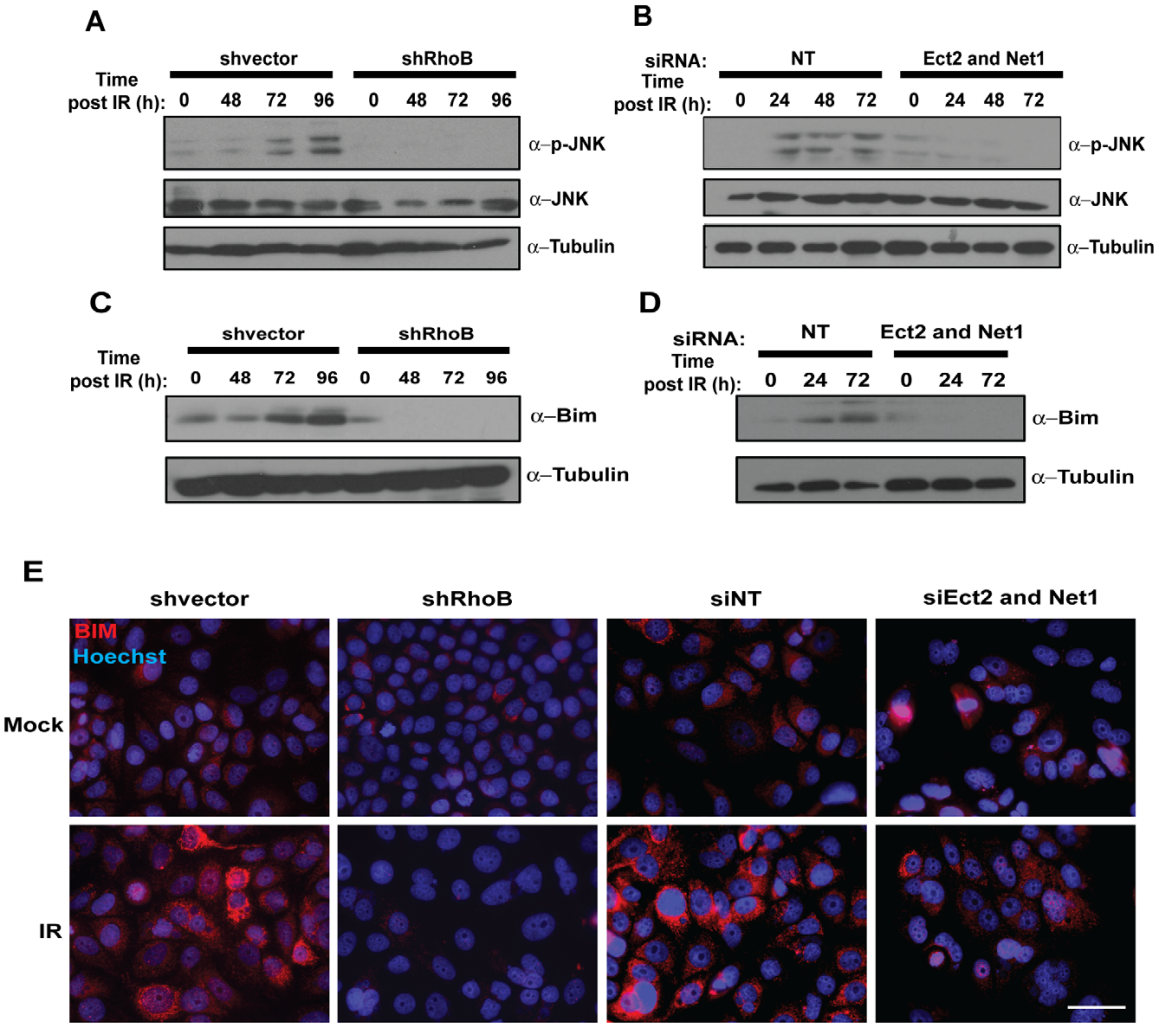
Loss of RhoB activity inhibits cell death pathways. (A) MCF-7-shvector and MCF-7-shRhoB cells were mock-treated or irradiated with 10 Gy. Whole-cell extracts were analyzed by immunoblot for changes in JNK phosphorylation, with total JNK, and α-Tubulin as loading controls at the indicated times post-irradiation. (B) MCF-7 cells were transfected with siNT or siEct2/Net1 and analyzed for JNK phosphorylation as in *(A)*. (C) MCF-7-shvector and MCF-7-shRhoB cells were mock-treated or irradiated with 10 Gy and analyzed for Bim protein expression at the indicated times. (D) MCF-7 cells transfected with siNT or siEct2/Net1 were mock-treated or irradiated with 10 Gy 48 h after transfection and analyzed for Bim protein expression at the indicated times. (E) Visualization of Bim induction by immunofluorescence in MCF-7-shvector, MCF-7-shRhoB cells or MCF-7 cells transfected with siNT or siEct2/Net1 that were mock-treated or irradiated with 10 Gy and fixed 72 h later. *Scale bar  = 50 µm*.

IR has been shown to induce apoptosis in a variety of cell types through the activation of c-Jun N-terminal kinase (JNK) and p38 mitogen-activated protein kinase (MAPK) [24]. We therefore wanted to determine if IR-induced cell death downstream of RhoB activation also involved the JNK pathway. MCF-7-shvector and MCF-7-shRhoB cells were irradiated, and lysates harvested at various times after IR were monitored for JNK phosphorylation by immunoblot analysis. MCF-7-shvector cells showed an increase in JNK activation as indicated by an increase in JNK phosphorylation as early as 48 h after IR (Fig. 5A). However, in MCF-7-shRhoB cells, JNK activation was dramatically suppressed, which correlates with the increase in survival of these cells (Fig. 5A and Fig. 2B). To test whether KD of Ect2 and Net1 exhibited a similar effect on the JNK pathway, MCF-7 cells were transfected with siNT or siEct2/Net1, irradiated 48 h after transfection and JNK phosphorylation examined 24–72 h later. MCF-7 cells transfected with siNT exhibited an increase in JNK phosphorylation that was suppressed by the transfection of siEct2/Net1 in a manner comparable to RhoB KD (Fig. 5B). However, knock-down of each GEF singly was not able to completely suppress JNK phosphorylation after IR, suggesting individually, Ect2 or Net1 may be able to compensate for one another (Fig. S4A).

Since we found that RhoB controls cell death upstream of JNK activation, we wanted to test if this pathway was functioning through the activation of Bim; a known JNK target. MCF-7-shvector and MCF-7-shRhoB cells were irradiated, and Bim protein levels monitored at various times after IR. The induction of the ∼23 kDa Bim isoform, Bim_EL_ was more pronounced in irradiated MCF-7-shvector cells then in MCF-7-shRhoB cells (Fig. 5C) and followed the kinetics of JNK activation with the most robust induction at 96 h (Fig. 5A and Fig. 5C). An increase in Bim immunofluorescence was also observed in MCF-7-vector cells, but not MCF-7-shRhoB cells after IR (Fig. 5E). Alone, siRNA-mediated KD of Ect2 or Net1 was not sufficient to inhibit increases in Bim_EL_ protein levels (Fig. S4B), however, KD of both Ect2 and Net1 was also able to decrease Bim_EL_ induction and Bim immunofluorescence compared to siNT control cells (Fig. 5D and 5E). These data illustrate that IR-induced activation of RhoB by the nuclear GEFs Ect2 and Net1 is necessary for the triggering of the apoptotic machinery involving JNK activation and Bim_EL_ induction.

## Discussion

**Figure 6:**
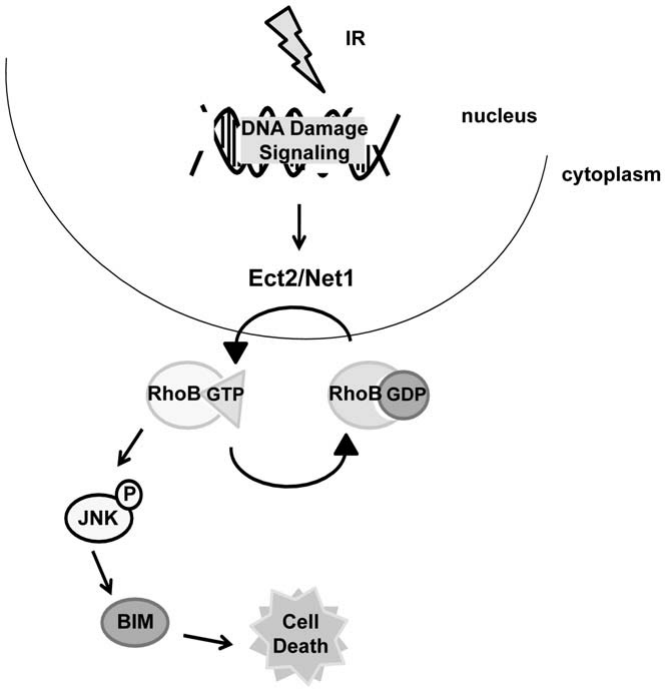
Model of RhoB-induced cell death after IR. IR-induced DNA damage activates members of the DNA damage-sensing machinery, which may directly or indirectly activate the nuclear GEFs Ect2 and Net1 to stimulate guanine nucleotide exchange upon RhoB. Activated RhoB leads to the downstream phosphorylation of JNK that triggers the induction of the pro-apoptotic protein Bim leading to cell death. Inhibition of either RhoB or Ect2 and Net1 activation through RNAi mutes this signaling pathway and results in cellular resistance to IR.

The exposure of eukaryotic cells to genotoxic agents results in a variety of cellular responses, which can either promote or prevent survival. Deciphering the signaling pathways that modulate these signals is of important clinical relevance as a prognostic indicator of tumor response to DNA damaging agents. It has been well established that the protein expression of the small GTPase RhoB is upregulated in response to cellular stress [9] and that RhoB is required to induce apoptosis after exposure to several DNA damaging agents in Ras-transformed cell lines [22]. In the present study, we further demonstrate that RhoB activity is increased upon exposure to DNA damaging agents, and this activity is necessary for IR-induced cell death. However, it is yet unclear what the upstream regulatory processes are that control RhoB activity after DNA damage. Therefore, we sought to elucidate these upstream modulators with specific focus on Rho GEFs. We show that the nuclear GEFs Ect2 and Net1 specifically activate RhoB, which causes the downstream phosphorylation of JNK and the induction of the pro-apoptotic protein Bim, leading to cell death (Fig. 6).

Of the ≥70 identified GEFs, Ect2 and Net1 specifically localize to the nucleus at steady state. Whereas Ect2 plays a physiologic role in cytokinesis [25], the normal functions of Net1 are still being defined. Here, we describe a new function for these GEFs in the modulation of cell death after genotoxic stress. Ect2 and Net1, out of the panel of GEFs tested, were the only two activated after DNA damage. Studies by others have shown Net1-mediated RhoA activation after exposure to supra-lethal doses (20 Gy) of IR [26]. However, under the lower doses of IR used in our studies, we did not observe increases in RhoA activity (Fig. 1E and 1F). In contrast to Ect2 and Net1, the cytoplasmic GEFs, p115 RhoGEF and Vav2 were not activated after IR (Fig. 3A). These results were not due to the inability of these GEFs to exchange upon RhoB, as overexpression of each GEF was sufficient to cause an increase in RhoB-GTP levels in cells (Fig. 3C). Moreover, despite the transient nature of protein KD, it is apparent from our data that KD of Ect2 and Net1 at the time of insult was sufficient to prevent the later (∼24 h) downstream activation of JNK (Fig. 5B, 5D–E) and to promote cell survival after IR (Fig. 4C) by muting RhoB-mediated death signals.

Our data demonstrate that IR activates both Ect2 and Net1, however the mechanisms that regulate their activity remain unknown. In many circumstances, GEF activation is linked to subcellular localization. Ect2 and Net1 are unique in this aspect as they both contain two nuclear localization signals within the N terminus, confining them to the nucleus at steady state. In addition to localization, GEF activation can be modulated by post-translational modification. For example, the phosphorylation of Ect2 during G_2_/M increases its activity for Rho *in vitro* [25] and the oncogenic form of Net1 was also shown to be regulated by its phosphorylation state [27]. It is therefore likely that specific kinase(s) may be responsible for their regulation and/or possible cellular relocation after DNA damage. The phosphoinositide 3-kinase-related protein kinase (PIKK) family consists of large serine/threonine protein kinases involved in the response to cellular stress, and are candidates for Ect2 and Net1 activation after IR. Members of this family include Ataxia-telangiectasia mutated (ATM), DNA dependent protein kinase catalytic subunit (DNA-PKcs), and ATM and Rad3 related protein kinase (ATR). ATM is an attractive candidate as it has a pivotal role in the response to IR-induced DNA damage. The association of ATM with other DNA damage related proteins or protein-complexes facilitate its activation and function. A number of the ATM-protein interactions occur with proteins which contain a BRCA-1 C-terminal (BRCT) domain; the most notable is Brca1 (breast cancer gene 1). The BRCT domain is a highly conserved region found in many DNA damage-responsive cell cycle checkpoint proteins [28]. Interestingly, Ect2 contains tandem BRCT domains in its N-terminus, which are required for proper cytokinesis [29]. It is therefore possible that these BRCT regions within Ect2 may have additional functions, some of which are more intimately involved in the DNA damage response. Net1, on the other hand, contains a nuclear export signal in addition to nuclear import signals, thus implying that it can be triggered to leave the nucleus and activate Rho in the cytoplasm [30]. To this effect, Schmidt and Hall have shown that the PH domain of Net1 is required for its nuclear export [30]. It is therefore of interest if DNA damage-mediated ATM activation triggers Ect2 and/or Net1 activation and relocation to the cytoplasm where it can activate RhoB. Preliminary data suggest that there is an enrichment of Ect2 in the cytoplasmic fraction after damage, and that loss of ATM kinase activity decreases Ect2 and Net1 activation after IR (data not shown). The cross-talk between ATM and these nuclear GEFs may be a mechanism for amplifying death signals in cells were irreversible damage occurs, and further studies are underway to delineate the mechanisms whereby DNA damage regulates Ect2 and Net1 function.

There are many circumstances where altered GEF activity can lead to cellular transformation [31]. In the case of Ect2 and Net1, truncation of the N-terminal portion of each protein has been shown to transform cells due to the mislocalization of these GEFs to the cytoplasm, which results in their constitutive activation [32], [33]. It would therefore be of interest to determine if the mislocalization of Ect2 and/or Net1 to the cytoplasm could be a potential resistance factor in human cancers to IR or other DNA damaging agents. Recently, Ect2 has been shown to be overexpressed and mislocalized to the cytoplasm of primary non-small cell lung carcinoma (NSCLC) tumor cells as well as NSCLC cell lines, but not primary normal lung epithelia [34]. These cells also have low levels of RhoB, and re-expression of RhoB decreases proliferation and tumor growth *in vivo* [3]. It is not surprising then, that the NSCLC cell line, A549 has been well studied for its radio-resistance [35]. Similarly, Ect2 is also overexpressed and mislocalized in glioblastoma multiforme (GBM) compared to normal brain tissue [36] and although GBMs respond to full course radiation, they tend to recur. It is therefore interesting to speculate that the mislocalization of Ect2 to the cytoplasm may be in part responsible for the relative radiation resistance and/or recurrence of these tumor cells.

RhoB plays a role in number of pathways which regulate tumor cell survival and proliferation. For example, it was demonstrated that RhoB controls endocytic trafficking and slows the internalization of the EGF receptor to the lysosome through PRK1 activation [37] as well as the regulation of nuclear Akt trafficking [38]. As important as RhoB is in modulating survival pathways little has been done to explore its role downstream of DNA damage. Some studies have shown that in response to farnesyl transferase inhibitor treatment, RhoB suppresses cyclin B1 leading to cell death [39] and it may associate with caspase-2 in mouse cardiomyocyte apoptosis [40]. In addition, RhoB represses NFκB activation after cell treatment with alkylating agents [41]. Therefore, we explored the pathways activated downstream of RhoB. We observed that the IR resistance of RhoB KD cells was not due to general alterations in the cell death machinery, as these cells were still capable of undergoing apoptosis after treatment with other cytotoxic agents such as staurosporine (unpublished results). Furthermore, RhoB deficient MEFs have an intact p53 response and undergo cell cycle arrest after IR [12]. These data suggest that RhoB may act downstream of the DNA damage response, functioning as a signal amplifier after cells have already been committed to die. Thus, we first examined the activation of JNK since it is commonly triggered after exposure to IR [24] and particularly in MCF-7 cells where it is needed to initiate apoptosis after IR as its inhibition prevents the release of cytochrome c [42]. Furthermore, overexpression of Rho family members A, B, and C can induce its activation [43]. We found JNK to be downstream of RhoB activation and that suppression of RhoB activity, either by reducing RhoB protein levels directly or reducing the amount of stimulating GEFs, Ect2 and Net1, was sufficient to inhibit JNK phosphorylation (Fig. 5A and 5B). Recently, JNK activation after IR was shown to be upstream of RhoB protein induction in Jurkat cells, however JNK activation downstream of RhoB was not examined [44]. Based on our own findings, there may exist a positive feedback loop whereby RhoB activity stimulates sustained JNK phosphorylation, which potentiates cell death through the upregulation of RhoB.

Of the many pro-apoptotic cellular targets of JNK, Bim can be activated transcriptionally via activation of the transcription factors such as c-Jun [45] and FOXO3a [46] or after translation in response to cytotoxic stimuli [47], [48]. We found that exposure to IR increases Bim_EL_ protein expression as early as 48 h, and that Bim protein levels are reduced by RhoB shRNA (Fig. 5C). Alternatively, if RhoB activity is inhibited through suppression of Ect2 and Net1, Bim_EL_ levels are also reduced (Fig. 5D), suggesting that it is increased RhoB activity, specifically, that is necessary for this event. Since JNK-dependent dephosphorylation and nuclear accumulation of FOXO3a were found in response to paclitaxel treatment [49], based on our own data, it is likely that RhoB-mediated Bim induction is primarily through a JNK-dependent mechanism.

Collectively, our experiments delineate a novel pathway whereby the nuclear GEFs Ect2 and Net1 are activated after genotoxic stress. These GEFs activate RhoB, which is required for cell death after exposure to γ-IR in non-Ras transformed human breast cancer cells. Furthermore, we demonstrate that RhoB activation triggers the downstream activation of the SAPK/JNK pathway leading to an increase in Bim protein levels and cell death. From a therapeutic standpoint, understanding the mechanisms of IR-induced cell death is of particular clinical relevance. Therefore, exploring the regulation of these nuclear GEFs after DNA damage may initiate novel strategies for rendering tumors which are refractory to radio- and/or chemotherapy more sensitive to first line anticancer treatments.

## Materials and Methods

### Cells, Culture Conditions, and Reagents

MCF-7 and HeLa cells were purchased from the American Type Culture Collection (Manassas,VA). A puromycin-selectable pLKO1 plasmid containing a short hairpin small interfering RNA against RhoB (shRhoB) or vector alone (shvector) control were used to infect MCF-7 cells (Open Biosystems, Huntsville, AL). Puromycin-resistant pooled populations were screened for RhoB protein expression and used to create stable cell lines. All cells were grown in high glucose-containing DMEM containing 5% fetal bovine serum (FBS), 0.5 µg/mL puromycin (for MCF-7 shvector, and MCF-7 shRhoB cells), 2 mM L-glutamine, penicillin (100 units/mL), and streptomycin (100 mg/mL) at 37°C in a 10% CO_2_, 90% air humidified atmosphere. All tissue culture components were purchased from Invitrogen (Carlsbad, CA). Cells were free of mycoplasma contamination. T-butyl peroxide (hydrogen peroxide, H_2_O_2_) (Sigma-Aldrich, St. Louis, MO) and human recombinant EGF (R&D Systems, Minneapolis, MN) were diluted in growth medium prior to treatment.

### Clonogenic Survival Assays and Irradiation

Survival was assessed by colony-forming ability using standard techniques [50]. Cells were irradiated using a Mark I ^137^Cs irradiator (JL Shepherd) as described [14] with the indicated doses and colonies assessed approximately 14–20 days after treatment. For siRNA experiments, cells were irradiated 72 h after transfection. Colonies were defined as >50 normal appearing cells originating from a single plated cell. Statistical significance was determined by a paired Student's *t-*test from a minimum of two separate experiments performed in triplicate and error bars represent the S.E.M.

### RBD and Nucleotide Free Pulldowns

RBD pulldowns to detect active Rho proteins were performed as described previously [18]. Affinity precipitation of active GEFs with the nucleotide-free Rho mutants (G17A) have been described in detail by our laboratory and performed as described with minor modifications [18], [19]. After lysis, samples were sonicated twice on ice for ∼30 sec bursts. Clarified lysates that were equalized for total volume and protein concentration were incubated with 20 µg of purified RhoB(17A) bound to glutathione-sepharose beads for 60 minutes at 4°C with rotation. Samples were washed in lysis buffer and processed for SDS-PAGE.

### Immunoblotting

SDS-PAGE gels were prepared as previously described [14]. Antibodies to pan-Rho (BD Biosciences, San Jose, CA), RhoB, Vav2 (Cell Signaling, Danvers, MA), RhoA (sc-418), Ect2 (sc-1005), p115 (sc-8492) (Santa Cruz Biotechnology, Santa Cruz, CA), and Net1 (Abcam, Cambridge, MA) were used for immunoblot analyses of RBD and nucleotide free pulldowns. To examine apoptotic proteolyses, cytoplasmic and nuclear extracts were prepared after IR. Briefly, cells were lysed in Buffer A [50 mM Hepes (pH 7.4), 10 mM KCl, 1 mM EDTA, 1 mM EGTA, 1 mM DTT, and 0.1% NP-40] plus 200 µM sodium orthovanadate and protease inhibitors. Samples were incubated on ice for 10 min with periodic vortexing and spun at 4000 rpm for 5 min. The cytosolic supernatant was equalized for total volume and protein concentration then processed for SDS-PAGE. Immunoblot analysis was performed using antibodies to phosphorylated JNK, total JNK (Cell Signaling), and Bim (Strategene, La Jolla, CA). For immunoblot quantification, intensity values of bands were measured from three different replicates for each experiment using Image J (NIH). The data were expressed as the fold increase over untreated samples. Statistical significance was determined by a paired Student's *t-*test and error bars represent the S.E.M.

### siRNA Oligonucleotides

Control siRNA oligonucleotides and those specific for human Net1 (targeted sequence: 5′-GAGUCUCCCUUCAGUCGAA-3′), Ect2 (targeted sequence: 5′-GCACUCACCUUGUAGUUGA-3′), and si*GENOME SMART*pool human RhoA and RhoB were purchased from Dharmacon (Lafayette, CO). Oligonucleotides were transfected using TransIT-siQUEST reagent, according to the manufacturer's instructions (Mirus Corporation, Madison, WI). Knock-down efficiency was determined for each experiment by immunoblot.

### Plasmid Transfections

Constructs to pEGFP-N1-onco-Vav2 [51], pCMV-Myc-p-115 RhoGEF (Invitrogen), pCMV-myc-Net1 [52], pCGN-hygro-ΔN-Ect2-DH/PH/C fused to an HA tag (kind gift from Channing Der, UNC), and pCMV-myc-RhoB [18] were transfected into cells using Lipofectamine Reagent (Invitrogen). 24 h after transfection, cells were lysed and RBD pulldown experiments performed. All constructs were confirmed by DNA sequencing.

### Immunofluorescence

MCF-7 cells were irradiated with 10 Gy and fixed 72 h thereafter as previously described [53]. Fixed cells were then incubated with a primary Bim antibody (Cell Signaling) and an AlexaFluor 594 secondary antibody (Molecular Probes, Eugene, OR). Nuclei were visualized by Hoechst 33258 staining. Images were collected using a x63 numerical aperture 1.4 oil immersion objective at 594 nm using a Zeiss axiovert 200 M microscope equipped with a Hamamatsu ORCA-ERAG digital camera and acquired using Metamorph Workstation (Universal Imaging Corp.). Images shown are representative of experiments done at least thrice.

## Supporting Information

Figure S1 **Genotoxic stress increases RhoB activity.** Knock-down efficiency of RhoA and RhoB siRNA. **(**A) HeLa cells were transfected with siRNA oligos to a non-targeting sequence or a sequence targeting RhoA or RhoB, respectively. 72 h after transfection, cells were harvested and relative levels of RhoA and RhoB protein expression were determined by immunoblot analysis. α-Tubulin served as a loading control. H_2_O_2_ treatment causes an increase in RhoB-GTP. HeLa cells were treated with increasing doses of H_2_O_2_ for 20 min, then processed for active GTP-bound Rho using a GST-RBD pulldown. (B) blotted for pan-Rho; (C) blotted for RhoB; (D) blotted for RhoA. Lysates served as loading controls and were blotted for Rho or α-Tubulin as indicated.
(TIF)

Figure S2 **5-FU causes an increase in RhoB activity that is partially required for cell death.** (A) MCF-7 cells were treated with the indicated doses of 5-FU for 72 h and pulldowns performed using GST-RBD to detect activated RhoB. (B) MCF-7 cells were treated with the indicated doses of 5-FU and survival determined by PI exclusion 72 h after drug exposure. The average from three experiments are shown. Values marked with asterisks are significant from control (*, *p*≤0.05).
(TIF)

Figure S3 **RhoB is not directly activated by ROS.** (A) HeLa cells expressing wild-type (wt) or C16/C20A myc-RhoB were serum-starved and treated with the indicated doses of H_2_O_2_ for 20 min and pulldowns performed with GST-RBD and blotted with an antibody to myc. (B) HeLa cells were treated with 500 µM H_2_O_2_ for the indicated times and GEF activation determined using a modified pulldown to detect binding to the RhoB(17A) mutant. Samples were blotted with antibodies against the indicated GEFs. (C) Relative increases in GEF activation were determined by densitometry of three independent experiments wherein controls were set to 1.0. The nuclear GEFs Ect2 and Net1 are activated after treatment with the DNA damaging agent 5-FU. (D and E), MCF-7 cells were treated with 400 µM 5-FU and pulldowns performed for activated GEFs using the RhoB(17A) mutant 72 h after drug treatment. Immunoblots from pulldowns and lysates were probed with antibodies to (D) Ect2 and (E) Net1.
(TIF)

Figure S4 **Knock-down of Ect2 or Net1 alone is insufficient to abrogate JNK phosphorylation or Bim induction after IR.** MCF-7 cells were transfected with a non-targeting siRNA (siNT), an siRNA targeted to Ect2 (siEct2), or an siRNA targeted to Net1 (siNet1) and either mock-treated or irradiated with 10 Gy 48 h later. Whole-cell extracts were analyzed by immunoblot for changes in (A) JNK phosphorylation at the indicated times post-irradiation, where total JNK and α-Tubulin levels served as loading controls or (B) Bim protein levels at the indicated times post-irradiation.
(TIF)

Materials and Methods S1 **Supplementary materials and methods.**
(DOC)
