# Supplementary figures and images for: The Nuclear Guanine Nucleotide Exchange Factors Ect2 and Net1 Regulate RhoB-Mediated Cell Death after DNA Damage
Source: PLoS One. 2011 Feb 23;6(2):e17108. doi: 10.1371/journal.pone.0017108 (PMC3044157; doi:10.1371/journal.pone.0017108)

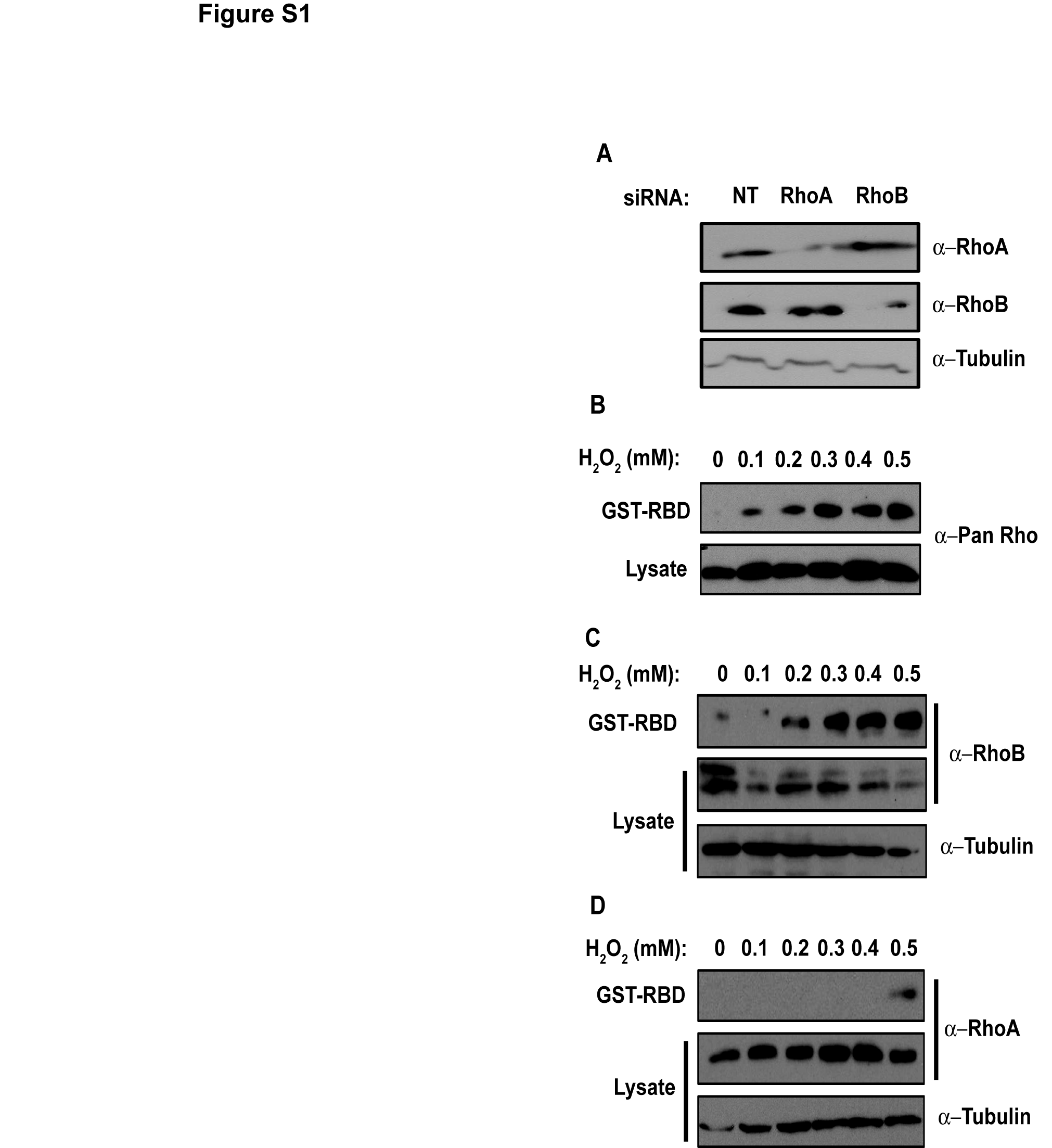

Supplement: Figure S1 — Genotoxic stress increases RhoB activity. Knock-down efficiency of RhoA and RhoB siRNA. (A) HeLa cells were transfected with siRNA oligos to a non-targeting sequence or a sequence targeting RhoA or RhoB, respectively. 72 h after transfection, cells were harvested and relative levels of RhoA and RhoB protein expression were determined by immunoblot analysis. α-Tubulin served as a loading control. H2O2 treatment causes an increase in RhoB-GTP. HeLa cells were treated with increasing doses of H2O2 for 20 min, then processed for active GTP-bound Rho using a GST-RBD pulldown. (B) blotted for pan-Rho; (C) blotted for RhoB; (D) blotted for RhoA. Lysates served as loading controls and were blotted for Rho or α-Tubulin as indicated. (TIF) [file pone.0017108.s001.tif]

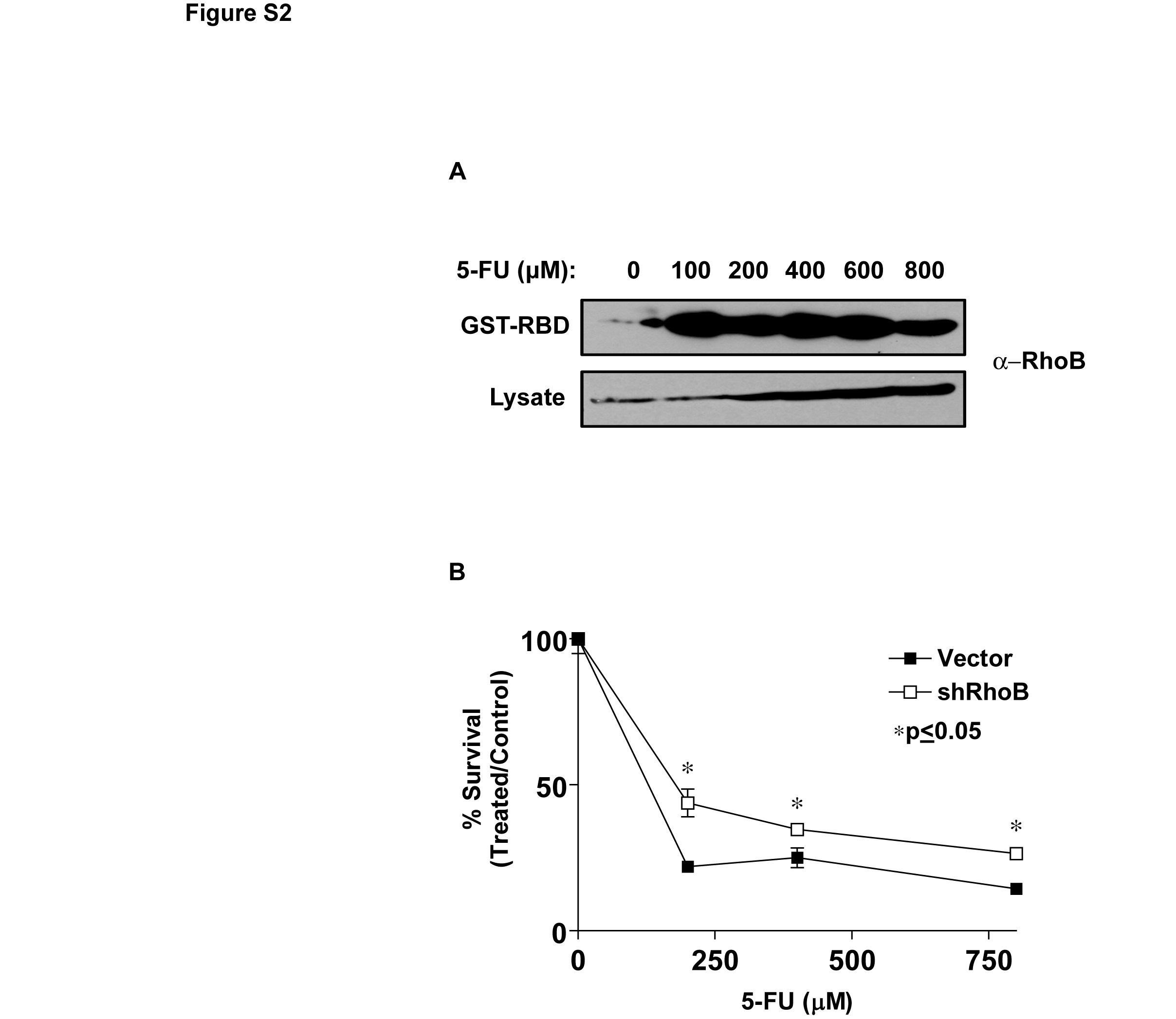

Supplement: Figure S2 — 5-FU causes an increase in RhoB activity that is partially required for cell death. (A) MCF-7 cells were treated with the indicated doses of 5-FU for 72 h and pulldowns performed using GST-RBD to detect activated RhoB. (B) MCF-7 cells were treated with the indicated doses of 5-FU and survival determined by PI exclusion 72 h after drug exposure. The average from three experiments are shown. Values marked with asterisks are significant from control (*, p≤0.05). (TIF) [file pone.0017108.s002.tif]

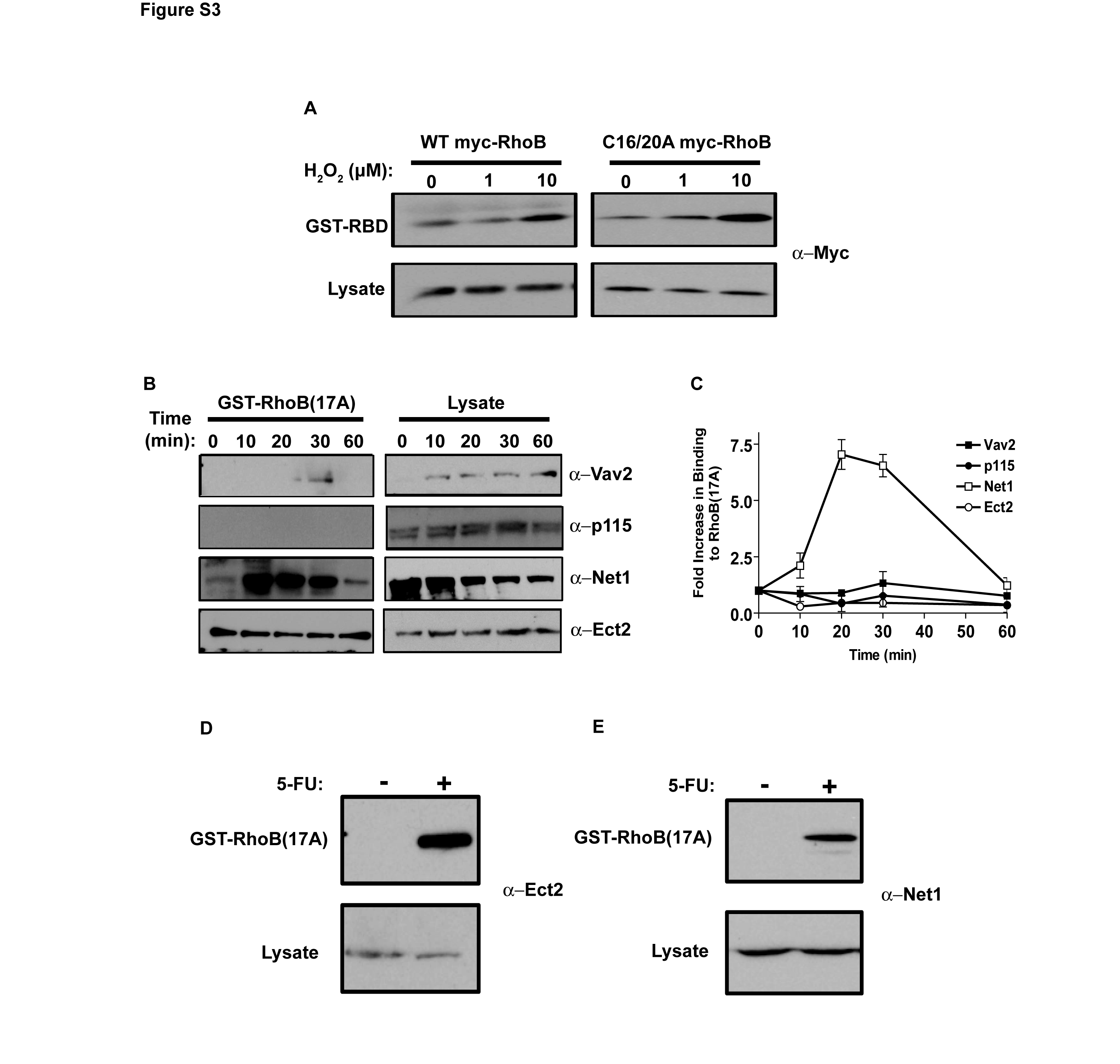

Supplement: Figure S3 — RhoB is not directly activated by ROS. (A) HeLa cells expressing wild-type (wt) or C16/C20A myc-RhoB were serum-starved and treated with the indicated doses of H2O2 for 20 min and pulldowns performed with GST-RBD and blotted with an antibody to myc. (B) HeLa cells were treated with 500 µM H2O2 for the indicated times and GEF activation determined using a modified pulldown to detect binding to the RhoB(17A) mutant. Samples were blotted with antibodies against the indicated GEFs. (C) Relative increases in GEF activation were determined by densitometry of three independent experiments wherein controls were set to 1.0. The nuclear GEFs Ect2 and Net1 are activated after treatment with the DNA damaging agent 5-FU. (D and E), MCF-7 cells were treated with 400 µM 5-FU and pulldowns performed for activated GEFs using the RhoB(17A) mutant 72 h after drug treatment. Immunoblots from pulldowns and lysates were probed with antibodies to (D) Ect2 and (E) Net1. (TIF) [file pone.0017108.s003.tif]

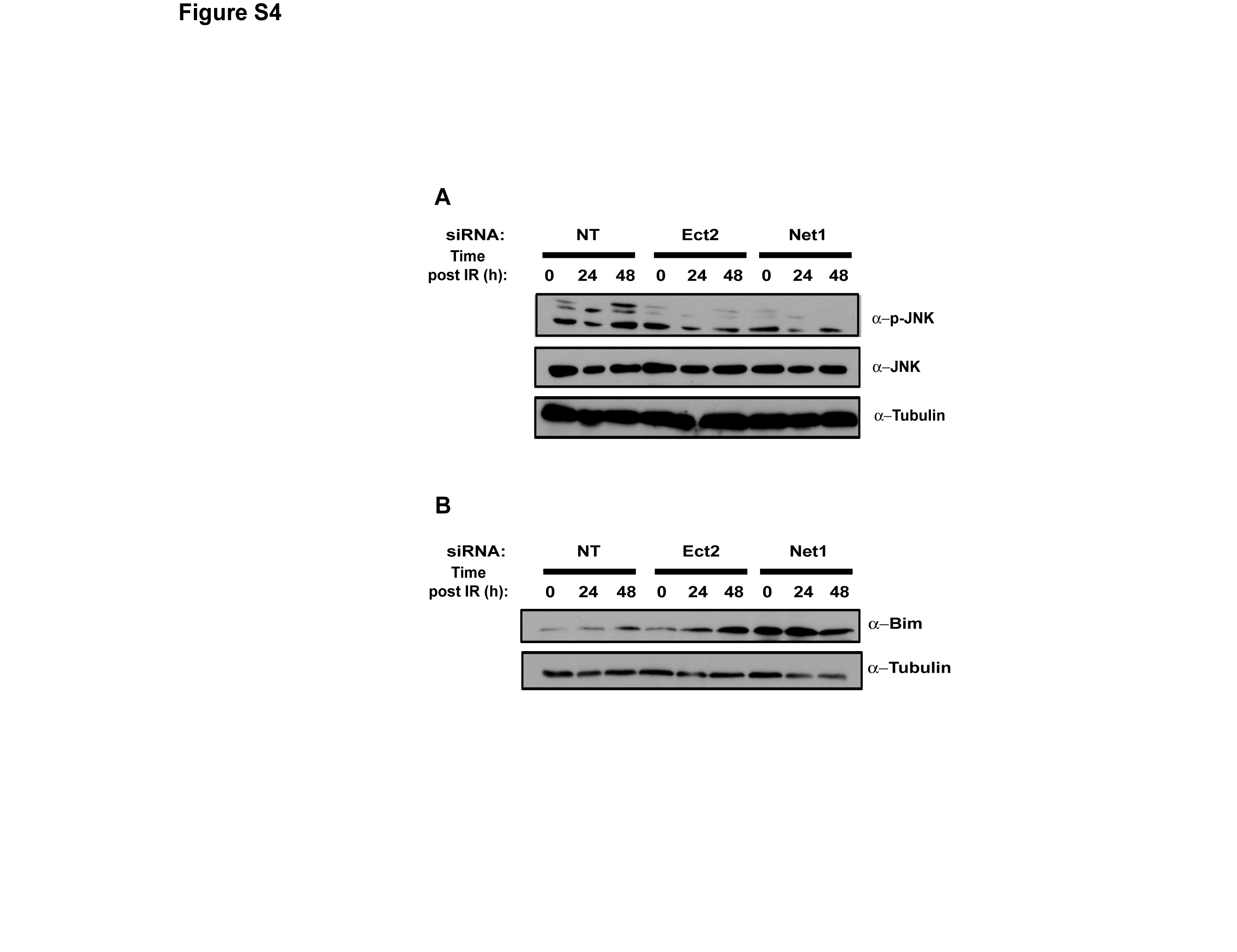

Supplement: Figure S4 — Knock-down of Ect2 or Net1 alone is insufficient to abrogate JNK phosphorylation or Bim induction after IR. MCF-7 cells were transfected with a non-targeting siRNA (siNT), an siRNA targeted to Ect2 (siEct2), or an siRNA targeted to Net1 (siNet1) and either mock-treated or irradiated with 10 Gy 48 h later. Whole-cell extracts were analyzed by immunoblot for changes in (A) JNK phosphorylation at the indicated times post-irradiation, where total JNK and α-Tubulin levels served as loading controls or (B) Bim protein levels at the indicated times post-irradiation. (TIF) [file pone.0017108.s004.tif]
