# Supplementary material for: The Nuclear Guanine Nucleotide Exchange Factors Ect2 and Net1 Regulate RhoB-Mediated Cell Death after DNA Damage
Source: PLoS One. 2011 Feb 23;6(2):e17108. doi: 10.1371/journal.pone.0017108 (PMC3044157; doi:10.1371/journal.pone.0017108)
Supplement: Materials and Methods S1 — Supplementary materials and methods. (DOC) [file pone.0017108.s005.doc]

**Supplementary Materials and Methods S1**

*Reagents-*5-Fluorouracil was purchased from Sigma-Aldrich, dissolved in DMSO and stored at -20 ˚C for no longer than three months.

*Generation of the RhoB cysteine mutant-* For generation of the RhoB cysteine to alanine mutant at C16 and C20, Rat wild-type RhoB in pCMV-myc was used as a template for site-directed mutagenesis (Stratagene). 24 h after transfection, cells were treated with the indicated doses of H_2_O_2_ for 20 min, lysed and RBD pulldown experiments performed.
